# An early prediction model for type 2 diabetes mellitus based on genetic variants and nongenetic risk factors in a Han Chinese cohort

**DOI:** 10.3389/fendo.2023.1279450

**Published:** 2023-10-25

**Authors:** Jinjin Li, Qun Ye, Hongxiao Jiao, Wanyao Wang, Kai Zhang, Chen Chen, Yuan Zhang, Shuzhi Feng, Ximo Wang, Yubao Chen, Huailin Gao, Fengjiang Wei, Wei-Dong Li

**Affiliations:** ^1^ Department of Genetics, College of Basic Medical Sciences, Tianjin Medical University, Tianjin, China; ^2^ NHC Key Laboratory of Hormones and Development, Tianjin Key Laboratory of Metabolic Diseases, Chu Hsien-I Memorial Hospital & Tianjin Institute of Endocrinology, Tianjin Medical University, Tianjin, China; ^3^ Center of Basic Medical Sciences, Tianjin Medical University, Tianjin, China; ^4^ Geriatric Medicine, Tianjin General Hospital of Tianjin Medical University, Tianjin, China; ^5^ Tianjin Nankai Hospital, Tianjin, China; ^6^ Institute of Laboratory Animal Sciences, Chinese Academy of Medical Sciences, Beijing, China; ^7^ Hebei Yiling Hospital, Shijiazhuang, Hebei, China

**Keywords:** type 2 diabetes mellitus, cohort study, genome-wide association study, Han Chinese, prediction model, genetic risk factors

## Abstract

**Aims:**

We aimed to construct a prediction model of type 2 diabetes mellitus (T2DM) in a Han Chinese cohort using a genetic risk score (GRS) and a nongenetic risk score (NGRS).

**Methods:**

A total of 297 Han Chinese subjects who were free from type 2 diabetes mellitus were selected from the Tianjin Medical University Chronic Disease Cohort for a prospective cohort study. Clinical characteristics were collected at baseline and subsequently tracked for a duration of 9 years. Genome-wide association studies (GWASs) were performed for T2DM-related phenotypes. The GRS was constructed using 13 T2DM-related quantitative trait single nucleotide polymorphisms (SNPs) loci derived from GWASs, and NGRS was calculated from 4 biochemical indicators of independent risk that screened by multifactorial Cox regressions.

**Results:**

We found that HOMA-IR, uric acid, and low HDL were independent risk factors for T2DM (*HR >*1; *P<*0.05), and the NGRS model was created using these three nongenetic risk factors, with an area under the ROC curve (*AUC*) of 0.678; high fasting glucose (FPG >5 mmol/L) was a key risk factor for T2DM (*HR* = 7.174, *P*< 0.001), and its addition to the NGRS model caused a significant improvement in *AUC* (from 0.678 to 0.764). By adding 13 SNPs associated with T2DM to the GRS prediction model, the *AUC* increased to 0.892. The final combined prediction model was created by taking the arithmetic sum of the two models, which had an *AUC* of 0.908, a sensitivity of 0.845, and a specificity of 0.839.

**Conclusions:**

We constructed a comprehensive prediction model for type 2 diabetes out of a Han Chinese cohort. Along with independent risk factors, GRS is a crucial element to predicting the risk of type 2 diabetes mellitus.

## 1 Introduction

Diabetes is a group of clinically and genetically heterogeneous diseases that are diagnosed by extraordinarily high blood glucose levels. It is a prevalent and rapidly growing noncommunicable chronic disease worldwide, with an expected increase in the number of affected adults from 2017 to 2045 of 50%, reaching a total of 693 million (1). In our country, approximately 92.4 million adults are already affected by diabetes (2), and approximately 90% of them have T2DM. It is well accepted that genetical and lifestyle factors contribute to T2DM (3). Numerous genetic studies have shown that there is a clear genetic predisposition to diabetes and its complexities (4). In recent years, researchers have identified more than 100 susceptibility genes and 200 susceptibility loci associated with the occurrence, development, and prognosis of T2DM by linkage analysis and large-scale GWASs (5), and the polygenic risk score calculated from these genes can predict the likelihood of developing T2DM (6). Sixty percent of the genes associated with T2DM found in Asian populations could be validated in Chinese populations (7). Hu et al. (8)confirmed the association of eight genes, namely *PPARG, KCNJ11, CDKAL1, CDKN2A-CDKN2B, IDE-KIF11HHEX, IGF2BP2*, and *SLC30A8*, with the prevalence of T2DM in a Chinese population study. Xu et al. (9) found that *CDKAL1* (rs7756992) and *SLC30A8* (rs13266634, rs2466293) were significantly associated with T2DM. In addition to genetic susceptibility, factors highly associated with the development of T2DM include age (10), obesity (11), lipid metabolism disorders (12), waist circumference (13), clinical biochemical indicators such as uric acid (14) and environmental factors such as lifestyle (15) and dietary habits (16).

Prediction of the risk of developing diabetes is important because of the large individual differences and the high number of complications. Diabetes models have been successfully established in some countries, such as the Framingham risk score diabetes model in the United States (17); the prediction model of diabetes onset in Mexican-descended Americans and non-Hispanic Caucasians by Stern (18); and the prediction model of diabetes onset risk in Japanese Americans by McNeely (19). There are two main T2DM models in China. Wu et al. counted the risk factors for diabetes onset in China over the past 20 years to establish the first T2DM risk assessment model for the Chinese population. In 2009, based on the Framingham cardiovascular prediction model, Chien et al. (20)established a T2DM risk prediction model for the Taiwanese population. The previous model only incorporated demographic indicators and laboratory measures of risk factors. With the development of GWAS, later models were built to include genetic factors as well, such as Meigs et al. (21) Framingham cohort for adding 18 SNPs as predictors.

Therefore, we need an early prediction model with high prediction value. Previous studies only showed a mild increase in *AUC* when SNPs were added to the prediction model. Although GWASs were performed in Han Chinese, many genes did not show high GRR due to low minor allele frequency (MAF) in Han Chinese. We conducted a prospective cohort study in a Han Chinese cohort, adding insulin resistance phenotypes and Chinese-specific SNPs to the prediction model.

## 2 Methods

### 2.1 Study design and population

The research was a prospective cohort that involved 297 participators from “The Tianjin Medical University Chronic Disease Cohort”. A total of 7,032 participants were recruited between 2006 and 2010, we selected samples that did not have diabetes in 2010 and had completed follow-up information up to 2015, then we coded and sorted these subjects by computer generated random numbers, and the top 305 people were chosen for genotyping. Follow-up was continued for further 4 years till 2019, with 8 people lost in follow-up, and the final number included in the analysis was 297. During the patient follow-up, 98 incident T2DM cases were identified, with a T2DM 9-year prevalence of 32.9%.

This study received approval from the Ethics Committee of Tianjin Medical University, and all participants signed informed consent forms.

### 2.2 Anthropometric measurements and biochemical indices

Anthropometric data, such as age, sex, weight, height, body mass index (BMI), and systolic/diastolic blood pressure (BP), were gathered. Laboratory examination: Blood samples were obtained via venipuncture in the morning after a 12-hour overnight fast, and measured by Hitachi automatic biochemical analysis. Fasting plasma glucose (FPG), serum creatinine (SCr), serum uric acid (SUA), blood urea nitrogen (BUN), C reactive protein (CRP), high-density lipoprotein (HDL), triglycerides (TG), total cholesterol (TC), total bilirubin (TBIL), total protein (TP), alanine aminotransferase (ALT), and fasting insulin (FINS) were measured at baseline. The homeostasis model assessment of insulin resistance (HOMA-IR) was computed employing the formula: (FPG (mmol/L) × FINS (mU/L)/22.5), and the quantitative insulin sensitivity check index (QUICKI) was computed employing the formula: 1/[log (FINS) (μU/ml) + log (FPG) (mg/dl)].

### 2.3 Diagnostic criteria

We defined diabetes as a fasting glucose level of 7 mmol/L or higher, or a two-hour glucose level of 11.1 mmol/L or higher and defined impaired fasting glucose as fasting glucose level of 6.1 to 6.9 mmo1/L (22). In accordance with the Chinese Hypertension Prevention Guide, hypertension was diagnosed based on a systolic blood pressure (SBP) ≥ 140 mmHg and/or diastolic blood pressure (DBP) ≥ 90 mmHg, or a history of hypertension (23). The diagnosis criteria for hyperuricemia were gender-specific, with males having a level of ≥ 420 µmol/L and females having a level of ≥ 360 µmol/L, excluding all drugs affecting uric acid metabolism (24).

### 2.4 Genotyping and SNPs selection

Blood samples were collected from all subjects using the high salt method to extract genomic DNA, which were subsequently genotyped using the Infinium Asian Screening Array-24 v1.0 BeadChip. After genotyping, systematic quality control analyses were carried out using PLINK 1.90 software (25): (i) Quality control procedures for genotypes: verifying the missingness rate of SNPs (>10%) and individuals with high missing rates (>5%); checking for difference in sex between the individuals recorded in the data and their sex based on X chromosome heterozygosity/homozygosity rates (the values for males and females should be >0.8 and<0.2, respectively); selecting autosomal SNPs with a MAF<0.05 and significant deviation from Hardy-Weinberg equilibrium (HWE) (*P<*1.0x10^−4^); identifying individuals who deviated ±3*SD* from the samples’ heterozygosity rate mean; and calculating the identicalness by descent (IBD) of all sample pairs, setting a pi-hat threshold of 0.2. (ii) Quality control for phenotypes: phenotypes included threshold traits (T2DM or not) and continuous diabetes-related traits (FPG, Hb1AC, insulin, HOMA-IR, QUICKI). The extreme values (values beyond the mean ±3*SD*) in the samples were excluded during quantitative trait correlation analysis. Thus, following the quality control procedures, 306659 SNPs and 273 samples were retained out of the initial 658849 SNPs and 297 samples for further association analyses.

### 2.5 Weighting approaches for constructing the wGRS and wNGRS

We developed GRS with selected highly correlated SNPs by genome-wide association analysis for T2DM-related phenotypes. We excluded SNPs that showed linkage disequilibrium (LD) with each other and analyzed the estimate by performing a logistic regression to determine the association between the number of risk alleles and T2DM.The weighted genetic risk score (wGRS) was calculated by multiplying the number of risk alleles (0, 1, or 2) for each SNP by the natural logarithm of the *OR* for that allele and summing across all SNPs, as described in formula (1). Similarly, the weighted nongenetic risk score (wNGRS) was calculated using the same principle as the wGRS. For each individual, the ωNGRS was calculated as the sum of risk factors weighted by the *HR* (*β*) value of different nongenetic risk factors in Cox regression, as described in formula (2). Assuming that genetic and nongenetic factors are independent, we added the weighted genetic score to each risk algorithm to obtain a combined nongenetic and genetic score. The comprehensive risk scoring model is the sum of the GRS and NGRS models, as described in formula (3).


$$\;\text{GRS}=\sum_{i=1}^{n}\text{βiGi}$$

Logit P(y=1|G)=α+GRS

(1) $$=\text{α}+\sum_{i=1}^{n}\text{βiGi}$$

βi is the weight of the ith SNP; Gi is the number of alleles at the ith SNP and assigns values of 0, 1, 2.


$$\text{ NGRS=}\sum_{i=1}^{m}\text{βiSi}$$

Logit P(y=1|S)=α+NGRS

(2) $$\text{ =α}+\sum_{i=1}^{m}\text{βiSi}$$

βi is the weight of the ith nongenetic risk factor. Si shows the status of the ith nongenetic risk factor, if the individual has the risk factor, the value is 1; if not, the value is 0.


Logit P(y=1|G, S)=α+GRS+NGRS

(3) $$=\text{α}+(\sum_{i=1}^{n}\text{βiGi}\text{ +}\sum_{i=1}^{m}\text{βiSi})$$

### 2.6 Power calculation

We performed power calculations using PASS 2021 (NCSS, LLC. Kaysville, Utah. http://www.ncss.com/software/pass/procedures/), using a two-sided test with α= 0.05. Of the 297 participants in our study, 98 were diagnosed with T2DM during the research period. Taking into account the prevalence of T2DM of 12.4% in China reported by Wang et al.(p0 = 0.124) (26), our sample size exhibited a power of 0.83 (e.g., OR=2.2 or less, depending on the distribution of the risk factor).

### 2.7 Statistical analysis

The SPSS26.0 statistical software package was employed for data analysis. Missing data imputation used the expectation-maximization algorithm (27). Continuous variables were compared utilizing either an independent samples t-test or a rank sum test, described by mean (standard deviation) or median (quartile) values, respectively. Categorical variables were compared utilizing the chi-square test. Independent risk factors were determined by Cox stepwise regression, *P*<0.05, and all differences were considered statistically significant. Additionally, genome-wide associations between diabetes-associated phenotypes and variation were examined using PLINK 1.9, and corresponding Manhattan and quantile-quantile plots were generated using the “manhattan” and “qqman” libraries in R (v.4.1.3) (28). The prediction model was constructed by logistic regression analysis, and utilized the AUC values to evaluate the predictive power of the model.

## 3 Results

### 3.1 Baseline clinical characteristics

**Figure 1:**
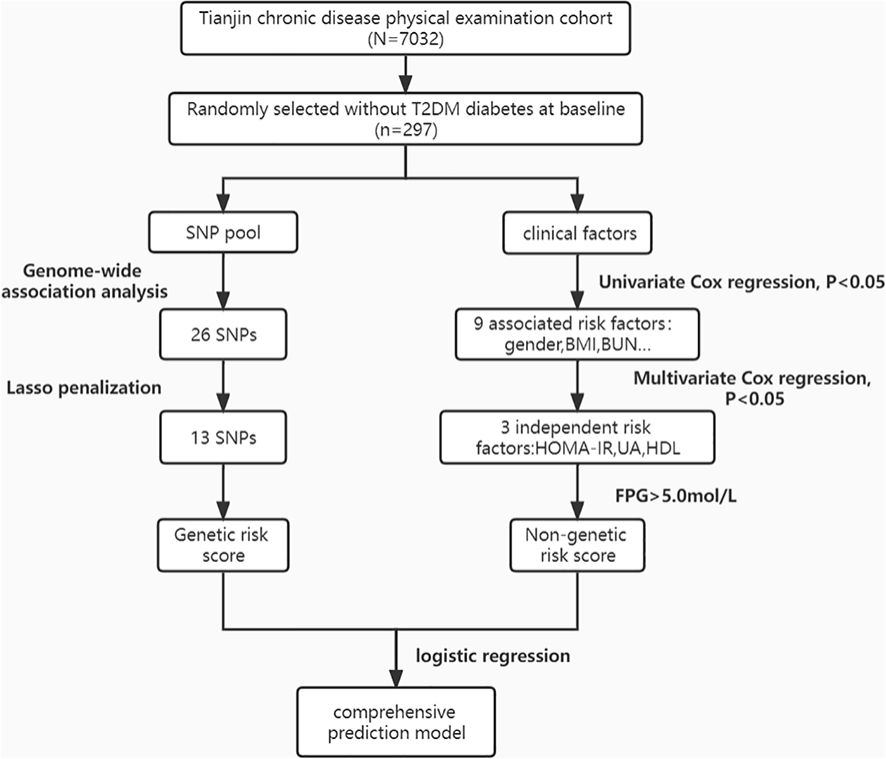
Flow chart of subjects in the prospective study.

The prospective research was conducted on 297 subjects (FPG< 7 mmol/L at baseline, age range 37–91) to establish a 9-year risk prediction model for T2DM (**Figure 1**). A total of 98 incident cases of T2DM, representing 32.9% of the study population, were identified. In our study, the mean age was 65.61 ± 13.55 years, and 59 subjects already had an impaired glucose test (fasting glucose 6.1-6.9mmol/L) at baseline, accounting for 19.9%, which may explain the higher prevalence of diabetes. Compared to the controls, the T2DM group had significantly higher levels of BMI, FPG, IFG, SUA, BUN, ALT, FINS and HOMA-IR. The T2DM group also had significantly lower levels of HDL and QUICKI. Although age, DBP, SCr, CRP, TG, TC, TBIL, and TP levels were higher in the T2DM group compared with the non-T2DM group, the differences were not statistically significant. **Table 1** shows the baseline characteristics of the study population.

**Table 1:** Baseline characteristics of participants with and without incident diabetes.

	Total(n=297)	Without incident diabetes (n=199)	With incident diabetes (n=98)	*t/χ^2^*	*P*
Men (%)	222(74.4%)	141(70.9%)	81(82.7%)	4.843	0.028
Age(years)	65.61 ± 13.55	64.88 ± 13.58	67.08 ± 13.44	-1.321	0.188
BMI (kg/m^2^)	24.71 ± 3.28	24.06 ± 3.13	26.04 ± 3.20	-5.07	<0.001
SBP10(mmHg)	135.7 ± 19.33	133.25 ± 18.43	140.68 ± 20.23	-3.159	<0.001
DBP10(mmHg)	72.48 ± 11.63	71.9 ± 11.88	73.66 ± 11.07	-1.232	0.219
FPG (mmol/L)	5.42 ± 0.67	5.12 ± 0.43	6.05 ± 0.620	15.129	<0.001
IFG (%)	59(19.9%)	7(3.5%)	52(53.1%)	101.25	<0.001
SCr(μmmol/L)	82.00 ± 19.00	82.00 ± 20.00	83.00 ± 19.00	-0.749	0.454
SUA(μmmol/L)	327.8372.59	312.19 ± 65.41	359.58 ± 76.32	-5.551	<0.001
BUN (mmol/L)	5.10 ± 1.60	4.90 ± 1.60	5.45 ± 1.90	-2.373	0.018
CRP (mg/L)	0.90 ± 1.10	0.90 ± 1.20	1.90 ± 1.10	-0.092	0.927
HDL (mmol/L)	1.34 ± 0.37	1.41 ± 0.39	1.21 ± 0.27	4.608	<0.001
TG (mmol/L)	1.35 ± 0.94	1.32 ± 0.95	1.40 ± 1.13	-1.061	0.289
TC (mmol/L)	4.86 ± 0.95	4.86 ± 0.98	4.86 ± 0.92	0.033	0.974
TBIL(μmol/L)	15.50 ± 5.20	15.00 ± 5.50	15.75 ± 4.90	-1.083	0.279
TP(g/L)	73.77 ± 4.24	73.63 ± 4.38	74.06 ± 3.95	-0.827	0.409
ALT (IU/L)	22.00 ± 12.00	21.00 ± 10.00	24.5 ± 11.00	-3.345	0.001
FINS (mU/L)	7.93 ± 6.19	7.46 ± 5.30	9.38 ± 7.41	-3.268	0.001
HOMA-IR	1.88 ± 1.61	1.66 ± 1.28	2.39 ± 2.01	-5.273	<0.001
QUICKI	0.61 ± 0.13	0.64 ± 0.13	0.58 ± 0.10	-5.335	<0.001

BMI, body mass index; SBP, systolic blood pressure; DBP, diastolic blood pressure; FBG, fasting plasma glucose; IFG: impaired fasting glucose; SCr, serum creatinine; SUA, serum uric acid; BUN, blood urea nitrogen; CRP, C-reactive protein; HDL, high-density lipoprotein; TG, plasma levels of triglyceride; TC, total cholesterol; TBIL, total bilirubin; TP, total protein; ALT, alanine aminotransferase; FINS, fasting insulin; HOMA-IR, homeostasis model assessment of insulin resistance; QUICKI, the quantitative insulin sensitivity check index.

### 3.2 Nongenetic risk factors for T2DM

**Figure 2:**
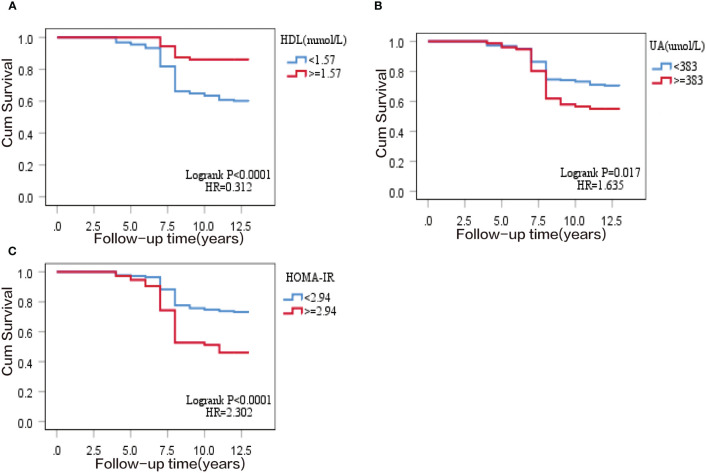
Kaplan–Meier survival curve of T2DM cumulative incidence in 297 subjects of the prospective study. **(A)** normal high value of homeostasis model assessment of insulin resistance (HOMA-IR); **(B)** normal high value of serum uric acid (SUA); **(C)** normal high value of high-density lipoprotein (HDL).

All variables (excluding collinear variables) with *P*<0.05 in the univariate model were involved in the multivariate model by gradual backward regression, with variable values<0.05 retained in the final model. Results from a Cox regression model revealed that SUA, HDL, and HOMA-IR were independent risk factors for T2DM. The regression coefficients of the factors retained in the final model are presented in **Table 2**. Additionally, Kaplan-Meier survival analyses revealed that higher quartile values of HOMA-IR, SUA, and HDL (defined as their normal high values) significantly impact T2DM onset in our study, as shown in (**Figure 2**).

**Table 2:** T2DM multivariate Cox regression analyses.

Variables	*β*	*SE*	*χ^2^*	*P*	*HR*	95% *CI*
SBP10	0.011	0.006	3.641	0.056	1.011	1.000-1.022
BMI	0.058	0.034	2.965	0.085	1.060	0.992-1.133
HDL	-0.842	0.365	5.318	0.021	0.431	0.211-0.881
SUA	0.004	0.002	5.676	0.017	1.004	1.001-1.007
HOMA	0.166	0.064	6.669	0.010	1.181	1.041-1.339
NGRS	1.882	0.441	18.230	<0.001	6.563	2.767-15.568

### 3.3 Results from genome-wide association studies

**Figure 3:**
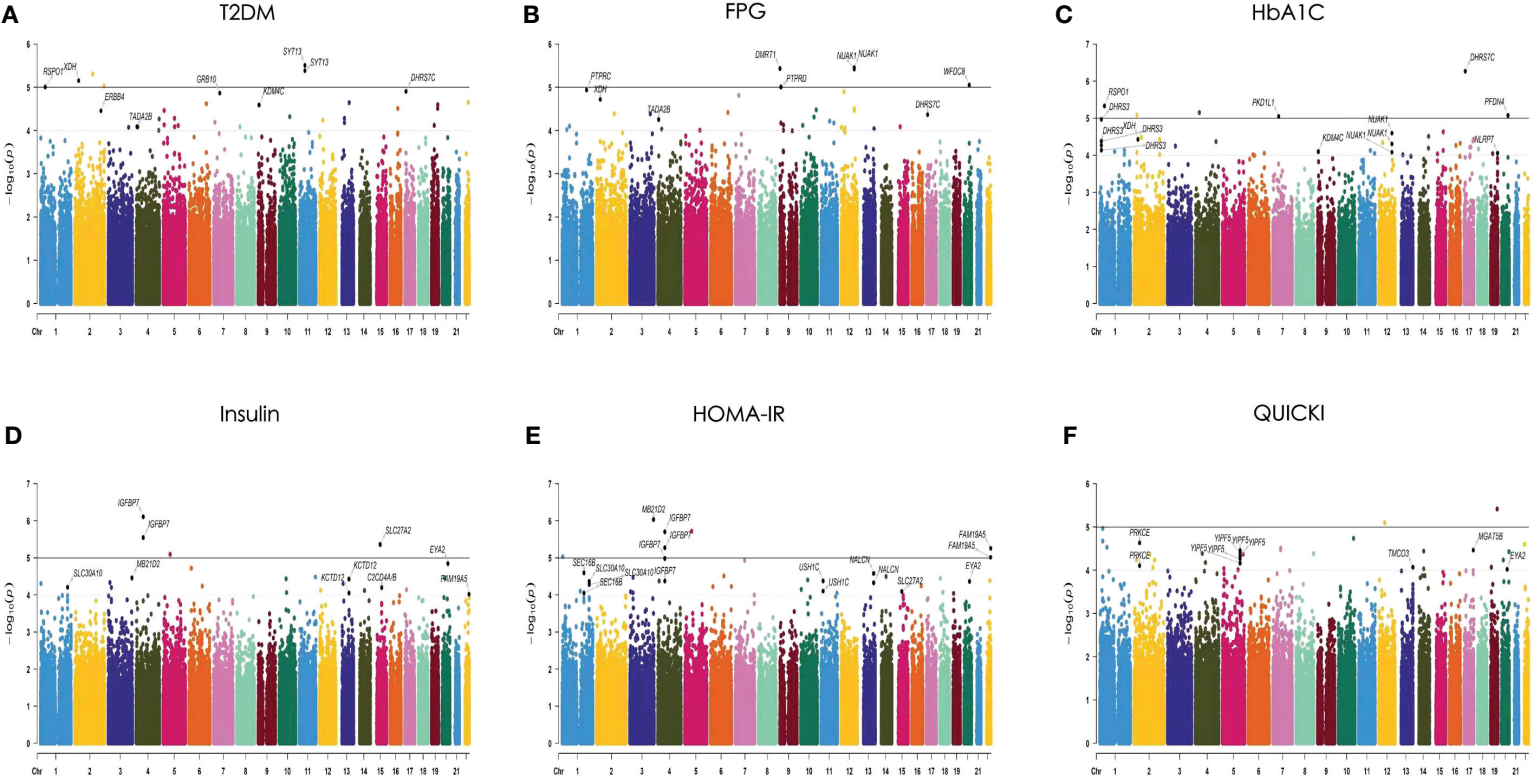
Manhattan plots of the P values for 6 traits in the generalized linear model (GLM) analysis. The 6 traits are **(A)** T2DM, **(B)** FPG, **(C)** HbA1C, **(D)** insulin, **(E)** HOMA-IR, and **(F)** QUICKI. The 22 chromosomes are shown in different colors. The solid line indicates the genome-wide significance level [−log10 (1× 10^−5^)]. The dashed line indicates the suggested significance level [−log10 (1 × 10^−4^)].

An analysis was performed on 306,659 autosomal SNPs that passed quality control to determine their association with six traits. Manhattan and QQ plots of the GWAS results are shown (**Figure 3**; **Supplementary Figure S1**). The analysis showed that no SNP reached the genome-wide significance threshold (P< 5× 10-8). Finally, we selected 26 SNPs from the T2DM-related phenotypes (T2DM, FPG, HbA1C, FINS, HOMA, QUICKI) based on *P*-values, SNP repeatability and biological significance of known mutations. Among them, rs10164462, rs_17_9691529, and rs76616810 were associated with T2DM, FPG, and HbA1C. rs8142739 was associated with insulin, HOMA-IR, and QUICKI. In addition, rs_3_192523400 rs11931598, rs17087830 rs16925187, rs1427793, rs_kgp4372010, and rs6066110 were all *P*< 1× 10^-4^ and associated with at least two T2DM-related traits (**Supplementary T** **able S1**). Some information of these SNPs, such as their genome locations, the closest reported genes, MAF and OR values, are exhibited in **Table 3**.

**Table 3:** Single-SNP association analysis of T2DM.

Chr	SNP	Nearby gene	Base-pair position	Genotype	EAF (Case/Control)	*OR* (*95%CI*)	*β*	*P*
1	rs_1_12637399	DHRS3	12637399	**C**/A	0.260/0.161	1.856(1.211-2.843)	0.618	1.08×10^-5^
1	rs76616810	RSPO1	38126486	**C**/T	0.133/0.035	4.467(2.221-8.986)	1.497	4.66×10^-6^
2	rs10164462	XDH	31552034	**A**/C	0.153/0.043	4.453(2.32-8.547)	1.494	7.01×10^-6^
2	rs_kgp9798346	ERBB4	212453661	C/**T**	0.806/0.644	2.385(1.555-3.658)	0.869	3.49×10^-5^
4	rs11931598	TADA2B	7047102	**C**/T	0.500/0.339	2.146(1.458-3.159)	0.764	5.57×10^-5^
5	rs62375492	YIPF5	143358522	**T**/C	0.296/0.214	1.599(1.064-2.405)	0.470	3.51×10^-5^
7	rs79535454	GRB10	50718600	**G**/A	0.101/0.023	4.964(2.178-11.312)	1.602	1.37×10^-5^
9	rs80314016	DMRT1	832245	**T**/C	0.196/0.101	2.325(1.393-3.879)	0.844	3.69×10^-6^
9	rs16925187	KDM4C	7043455	**G**/C	0.148/0.056	2.800(1.561-5.023)	1.030	2.57×10^-5^
9	rs1547287	PTPRD	9354303	C/**T**	0.776/0.626	2.109(1.406-3.164)	0.746	9.91×10^-6^
11	rs4755984	SYT13	45441337	**C**/T	0.515/0.304	2.293(1.611-3.262)	0.830	4.13×10^-6^
12	rs1427793	NUAK1	106458238	**G**/A	0.102/0.03	3.996(1.863-8.569)	1.385	3.52×10^-6^
17	rs_17_9691529	DHRS7C	9691529	**T**/A	0.25/0.121	2.617(1.627-4.211)	0.963	5.44×10^-7^
	GRS					2.796(2.210-3.536)	1.028	

Chr, chromosome; SNP, single nucleotide polymorphism; OR, odds ratio; EAF, effect allele frequency; GRS, genetic risk score; bold indicates effect allele.

### 3.4 Nongenetic risk score prediction model for T2DM

**Figure 4:**
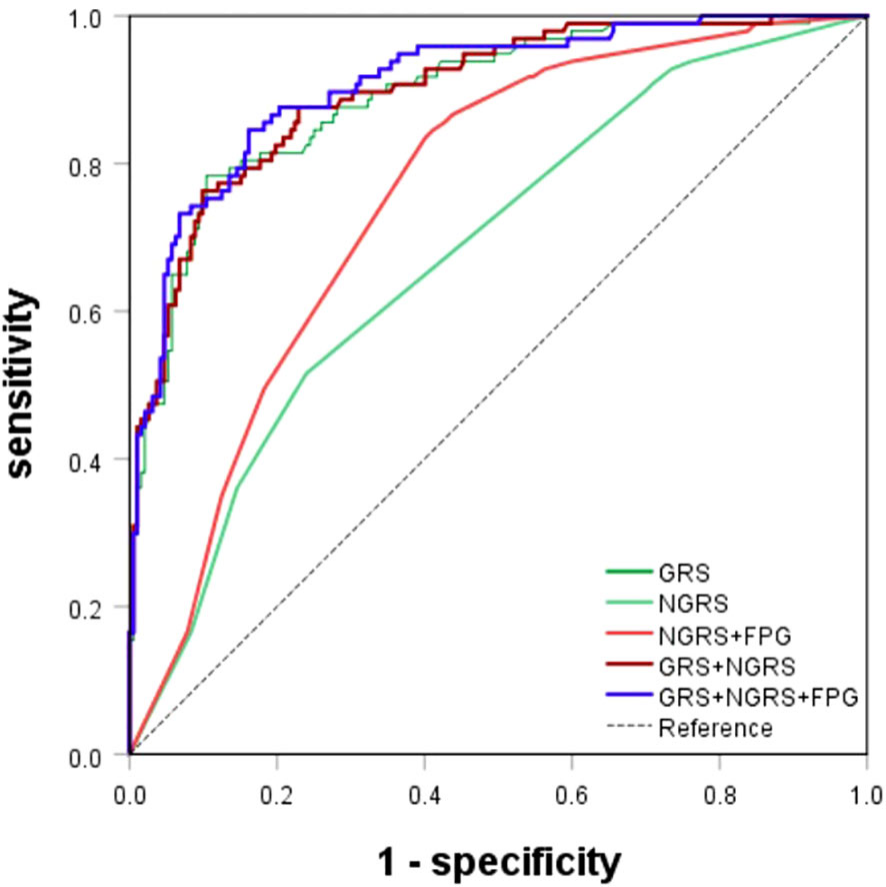
ROC curves for T2DM prediction model. The genetic (GRS), nongenetic (NGRS; NGRS+FPG), and comprehensive models (GRS+NGRS; GRS+NGRS+FPG).

The nongenetic prediction model for T2DM included normal high values of HOMA-IR, SUA, and HDL. The model showed a C statistic of 0.678 (95% CI: 0.614–0.742), with a sensitivity of 0.52 and a specificity of 0.764. The OR value was 6.563 (95% CI: 2.767-15.568) (**Table 4**; **Figure 4**). The prediction equation was logit P = -0.504 + (0.166 × S1 + 0.004 × S2+ (-0.842) × S3), while S1 = HOMA-IR normal high value (0:< 2.94; 1: ≥ 2.94), S2 = SUA normal high value (0: < 383 μmol/L; 1: ≥ 383 μmol/L), S3 = HDL normal high value (0: < 1.57 mmol/L; 1: ≥ 1.57 mmol/L). On the basis of the above optimized nongenetic risk factors, fasting blood glucose factors were added; S4 = FPG normal high value (0: FPG ≤ 5 mmol/L; 1: FPG > 5 mmol/L), the AUC was 0.764 (95% CI: 0.709-0.818), with corresponding sensitivity and specificity values of 0.837 and 0.608, respectively. The OR value was 3.183 (95% CI: 2.225-4.552) (**Table 4**; **Figure 4**).

**Table 4:** Logistic regression analysis and prediction power comparison of nongenetic (NGRS), genetic (GRS), and comprehensive models for T2DM.

model	Logistic regression analysis		ROC curve			
	*OR (95% CI)*	*P*	*AUC* (95% *CI*)	*P*	sensitivity	specificity
GRS^a^	2.796(2.210-3.536)	<0.001	0.892(0.853-0.932)	<0.001	0.784	0.896
NGRS^b^	6.563(2.767-15.568)	<0.001	0.678(0.614-0.742)	<0.001	0.520	0.764
NGRS+FPG^c^	3.183(2.225-4.552)	<0.001	0.764(0.709-0.818)	<0.001	0.837	0.608
GRS+NGRS^d^	2.803(2.210-3.555)	<0.001	0.898(0.860-0.936)	<0.001	0.763	0.901
GRS+NGRS+FPG^e^	2.473(2.008-3.045)	<0.001	0.908(0.872-0.944)	<0.001	0.845	0.839

OR, odds ratio, CI, confidence interval, AUC, area under curve, NGRS, nongenetic risk score model, GRS, genetic risk score model.

### 3.5 Genetic risk score prediction model for T2DM

Genetic prediction models for predicting the onset of T2DM use a weighted risk score approach, which can reveal the polygenic contribution to T2DM risk of SNPs that show disease association but falling short of the genome-wide significance threshold. The 26 SNPs associated with T2DM were identified by GWAS. Using the lasso penalty method, 13 SNPs were eventually included in the prediction model (**Supplementary Figure S2**). The GRS model yielded an AUC of 0.892 (95% CI: 0.853-0.932), a sensitivity of 0.784, a specificity of 0.896, and an OR of 2.796 (95% CI: 2.210-3.536) (**Table 4**; **Figure 4**). The genetic risk prediction equation logit P = -6.597 + (0.618 × rs_1_12637399Gi + 1.497 × rs76616810Gi + 1.494 × rs10164462Gi + 0.869 × rs_kgp9798346Gi + 0.764 × rs11931598Gi + 0.47 ×rs62375492Gi + 1.602 × rs79535454Gi + 0.844 × rs80314016Gi + 1.03 × rs16925187Gi + 0.746 × rs1547287Gi + 0.83 × rs4755984Gi + 1.385 × rs1427793Gi + 0.962 × rs_17_9691529Gi).

### 3.6 Comprehensive prediction model for T2DM

Upon evaluation and screening, the ultimate comprehensive predictive model is obtained by adding the arithmetic sum of the two models to the fasting glucose high value. It was logit P = -7.156 + (0.618 × rs_1_12637399Gi + 1.497 × rs76616810Gi + 1.494 × rs10164462Gi + 0.869 × rs_kgp9798346Gi + 0.764 × rs11931598Gi + 0.47 × rs62375492Gi + 1.602 × rs79535454Gi + 0.844 × rs80314016Gi + 1.03 × rs16925187Gi + 0.746 × rs1547287Gi + 0.83 × rs4755984Gi + 1.385 × rs1427793Gi + 0.962 × rs_17_9691529Gi + 0.166 × S1 + 0.004 × S2 + (-0.842) × S3 + 1.970 × S4). The comprehensive T2DM prediction model had a higher predictive value compared to either the nongenetic or genetic prediction models, with an AUC of 0.908 (95% CI: 0.872-0.944), an OR of 2.473 (95% CI: 2.008-3.045), a sensitivity of 0.845, and a specificity of 0.839 (**Table 4**; **Figure 4**). Additionally, the Hosmer-Lemeshow test indicated good calibration ability of the T2DM prediction model (*χ^2^* ^^= 11.191, *P* = 0.191).

### 3.7 Internal validation

Internal validation of different prediction models was carried out by using bootstrap ten-fold cross validation method. In this study, the AUC values verified by genetic (a), non-genetic (b; c) and comprehensive prediction (d; e) models were 0.872, 0.670, 0.734, 0.873, and 0.887, respectively, after 50 times of 10-fold cross-validation of different prediction models. The results show that the prediction model has good stability.

### 3.8 External validation

We used the Framingham Diabetes Risk Score to assess the risk of developing T2DM in the Chinese Han population in this study. The Framingham Diabetes Risk Score simple clinical model includes 9 indicators, including age, sex, BMI, family history of diabetes, SBP/DBP, HDL, TG, FPG, and waist circumference (17). This research lacks information on family history of diabetes and waist circumference. When the Framingham diabetes risk prediction model was applied to our study population, the AUC was 0.889 (95% CI: 0.847-0.931). However, the Framingham diabetes risk score uses a cut-off of FPG >5.5 mmol/L, whereas if we use the same cut-off as our model (FPG >5mmol/L), the AUC drops to 0.761 (95% CI: 0.707-0.815) (**Supplementary Figure S3**).

## 4 Discussion

Most of the existing studies in China have used only traditional laboratory indicators to construct diabetes prediction models, and few studies have used genetic risk factors as predictors. The combined use of SNPs to predict the risk of T2DM has been reported in other countries (29–31), and their genetic factors alone predicted an *AUC* between 0.55 ~ 0.6, traditional risk factors predicted an *AUC* of approximately 0.65 ~ 0.78, and the combination of both predicted an *AUC* of approximately 0.68 ~ 0.8. Therefore, there is a need to develop T2DM prediction models that include genetic risk factors in China. The AUCs of our genetic, nongenetic and combined risk prediction models were 0.892, 0.764 and 0.908, respectively. All three results were higher than those of other studies, indicating better predictive validity. Compared to other models, our model is unique in that it contains SNPs that are not common in European populations, and the model has Han-specific markers, which may be one of the reasons for the better performance of our model. By adding our genotyping data, the prediction model *AUC* was significantly improved (from 0.764 to 0.908).

This study included new phenotypic detections, such as FINS, HOMA-IR, and QUICKI, with HOMA-IR being an independent predictor of T2DM. In addition, some new genetic loci were identified as follows: rs4755984 in the *SYT13* gene, rs1547287 in the *PTPRD* gene, rs76616810 in the *RSPO1* gene, rs16925187 in the *KDM4C* gene, rs_kgp9798346 in the *ERBB4* gene, rs79535454 in the *GRB10* gene, rs1427793 in the *NUAK1* gene, rs62375492 in the *YIPF5* gene, and rs10164462 in the *XDH* gene. We found that the nearest genes to the above SNP loci were associated with metabolism or diabetes. The *SYT13* gene, located on chromosome 11, is a member of a large family of synaptic binding proteins. Compared to healthy adults, SYT13 gene expression is downregulated in T2DM patients, and downregulation of this gene decreases islet secretory function and is negatively associated with HbA1c levels *in vivo (* 32). SNP rs154738, located in the intron of *PTPRD*, had a less significant association with T2DM (*P* = 9.91 × 10^-6^; *OR* = 2.109, *95% CI* = 1.406-3.164). A previous GWAS of T2DM in a Han Chinese population identified PTPRD as a susceptibility gene for T2DM (33). Overexpression of PTPRD in preadipocytes (3T3L1) inhibits adipogenesis, but this may lead to the development of adipose ectopic accumulation and insulin resistance, favoring the development of T2DM. Additionally, in human subjects, a positive correlation was observed between serum RSPO1 levels and fasting C-peptide levels, which is a marker of insulin secretion. RSPO1 levels also presented a positive correlation with both obesity and insulin resistance (34). Also associated with obesity is *KDM4C* located at 9p24.1, a member of the JMJD2 family that promotes preadipocyte differentiation by repressing PPARγ transcriptional activation (35). Latorre (36) et al. found that *ERBB4*, located at 2q34, had significantly increased expression in the organs of obese people. Although these genes are not directly related to the development of T2DM, approximately 90% of T2DM patients are overweight or obese, and obesity caused by disorders of lipid metabolism is also considered important risk factor for T2DM development. The variants of *GRB10*, which is an inverse regulator of insulin signaling, have been shown to have a significant association with impaired β-cell function (37). In 2020, Franco (38) et al. identified *YIPF5* mutations as a major cause of monogenic diabetes. *XDH* (*XOR*), the rate-limiting enzyme produced by SUA, is not only highly expressed in hyperuricemia and gout but has also been shown to have significantly higher XOR activity in diabetic patients than in normal adults (39).

In addition, when including the glucose factor FPG>5 mmol/L, the *AUC* value of our prediction model in this study was 0.764; the Framingham diabetes risk prediction model had an *AUC* value of 0.761 for FPG>5 mmol/L and 0.889 for FPG>5.5 mmol/L. Although FPG>5.5 mmol/L may be a better T2DM “predictor”, it cannot achieve early prediction, and we should not use it in early prediction models.

The present study also has some limitations. First, all study participants were monitored for at least 4 years, but it is unclear whether they developed diabetes in the first 3 years due to missing data for the period from 2011 to 2013. In addition, we did not perform OGTT screening for those 297 subjects. Since OGTT requires multiple blood draws and patients have a low degree of cooperation at annual physical examinations, the diagnostic criteria for T2DM in our article is based on fasting blood sugar ≥7.0 mmol/L. The use of a single glucose measure as an outcome diagnostic criterion may overestimate the prevalence of T2DM, which is one of the limitations of most epidemiological studies. Third, genetic risk factors were selected from a relatively small sample size, and some potential bias exists in the study results. Lastly, to enhance the applicability of our model to other populations, further external validation in larger and younger cohorts is needed. We plan to conduct such studies in the future to refine and validate our T2DM prediction model.

## 5 Conclusions

Our study provides a comprehensive and accurate prediction model for T2DM risk, highlighting the importance of considering both traditional risk factors and genetic factors in disease prediction. The identification of novel genetic loci associated with T2DM risk also adds to our understanding of the underlying biology of this disease, potentially opening up new avenues for therapeutic intervention and disease prevention.

## Data availability statement

The variant data for this study have been deposited in the European Variation Archive (EVA) at EMBL-EBI under accession number PRJEB67630 (https://www.ebi.ac.uk/eva/?eva-study=PRJEB67630).

## Ethics statement

This study received approval from the ethics committee of Tianjin Medical University, and all participants signed informed consent forms. The studies were conducted in accordance with the local legislation and institutional requirements. The participants provided their written informed consent to participate in this study.

## Author contributions

JL: Data curation, Formal Analysis, Investigation, Methodology, Resources, Writing – original draft, Writing – review & editing. QY: Formal Analysis, Investigation, Methodology, Writing – original draft, Writing – review & editing. HJ: Investigation, Methodology, Software, Writing – review & editing. WW: Formal Analysis, Investigation, Writing – review & editing. KZ: Data curation, Investigation, Resources, Writing – review & editing. CC: Data curation, Investigation, Resources, Writing – review & editing. YZ: Formal Analysis, Investigation, Writing – review & editing. SF: Data curation, Investigation, Resources, Writing – review & editing. XW: Data curation, Resources, Writing – review & editing. YC: Methodology, Software, Writing – review & editing. HG: Data curation, Funding acquisition, Resources, Writing – review & editing. FW: Conceptualization, Investigation, Methodology, Supervision, Writing – review & editing. W-DL: Conceptualization, Funding acquisition, Investigation, Supervision, Writing – original draft, Writing – review & editing.
