# Supplementary material for: An early prediction model for type 2 diabetes mellitus based on genetic variants and nongenetic risk factors in a Han Chinese cohort
Source: Front Endocrinol (Lausanne). 2023 Oct 25;14:1279450. doi: 10.3389/fendo.2023.1279450 (PMC10634500; doi:10.3389/fendo.2023.1279450)
Supplement: Supplementary file 1 [file DataSheet_1.pdf]

Supplementary material

Table S1. Genome-wide association analysis with glucose metabolism-related indicators.
Figure S1. Quantile-quantile plot of the *P* values. The negative logarithm of the *P* value in the genome-wide association study using the generalized linear model (GLM) method for 6 T2DM-related quantitative traits are plotted against their expected values under the null hypothesis.
Figure S2. The LASSO regression algorithm was used to select the optimal variable (λ) with a 10-fold cross-validation method.
Figure S3. Area of *ROC* curve predicted by Framingham diabetes risk score for different fasting glucose thresholds. The blue curve is the predicted area of the model incorporating FPG>5.5; the red curve is the predicted area of the model incorporating FPG>5.

Table S1. Genome-wide association analysis with glucose metabolism-related indicators

CHR	SNP	Nearest gene	T2MD (*P* value)	FPG (*P* value)	HbA1c (*P* value)	Insulin (*P* value)	HOMA-IR (*P* value)	QUICKI (*P* value)
1	rs_1_12637399	DHRS3			1.08×10^{-5}			
1	rs76616810	RSPO1	9.92×10-6	9.58×10-5	4.66×10-6			
1	rs633715	SEC16B					2.54×10-5	
1	rs_1_219922629	SLC30A10				6.20×10-5	4.31×10-5	
2	rs10164462	XDH	7.01×10-6	1.91×10-5	3.77×10-5			
2	rs_2_45941401	PRKCE						2.34×10-5
2	rs_kgp9798346	ERBB4	3.49×10-5					
3	rs_3_192523400	MB21D2				3.47×10-5	9.22×10-7	
4	rs11931598	TADA2B	8.18×10-5	5.57×10-5				
4	rs17087830	IGFBP7				2.85×10-6	5.32×10-6	
5	rs62375492	YIPF5						3.51×10-5
7	rs79535454	GRB10	1.37×10-5					
9	rs80314016	DMRT1		3.69×10-6				

9	rs16925187	KDM4C	2.57×10-5		8.00×10-5			
9	rs1547287	PTPRD		9.91×10-6				
11	rs2072225	USH1C					4.22×10-5	
11	rs4755984	SYT13	4.13×10-6					
12	rs1427793	NUAK1		3.52×10-6	2.55×10-5			
13	rs17066095	KCTD12				8.94×10-5		
13	rs_13_114165266	TMCO3						8.58×10-5
15	rs_kgp4372010	SLC27A2				4.35×10-6	8.10×10-5	
15	rs7167881	C2CD4A/B				6.36×10-5		
17	rs_17_9691529	DHRS7C	1.25×10-5	4.30×10-5	5.44×10-7			
17	rs73362039	MGAT5B						3.43×10-5
20	rs6066110	EYA2				1.42×10-5	4.33×10-5	
22	rs8142739	FAM19A5				9.63×10-5	9.72×10-6	9.59×10-5

T2DM related gene loci were selected based on: (1) loci with $P < 1\times 10^{-4}$ and recurrence in association analysis of T2DM related quantitative traits ;(2) Consider the physical location distribution of SNP loci in genes. Focus on known biological significance such as mutations that have been reported to be associated with obesity, metabolism diseases

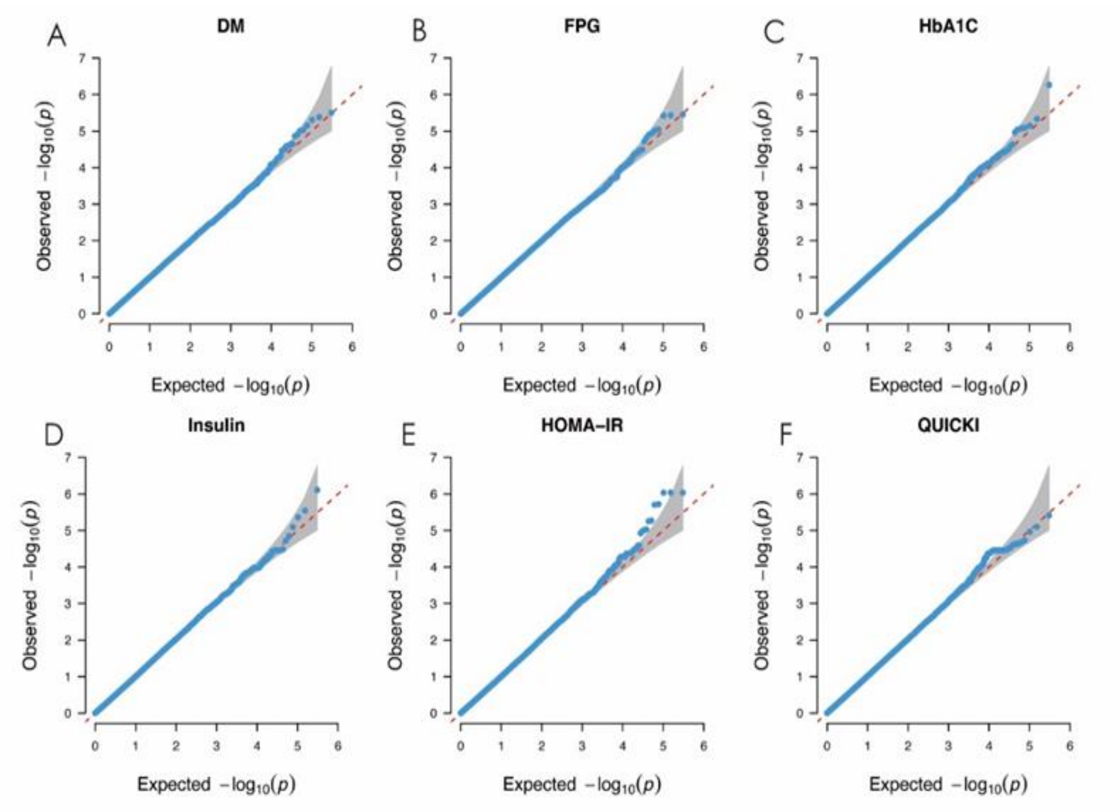

Figure S1. Quantile-quantile plot of the *P* values. The negative logarithm of the *P* value in the genome-wide association study using the generalized linear model (GLM) method for 6 T2DM related quantitative traits are plotted against their expected values under the null hypothesis

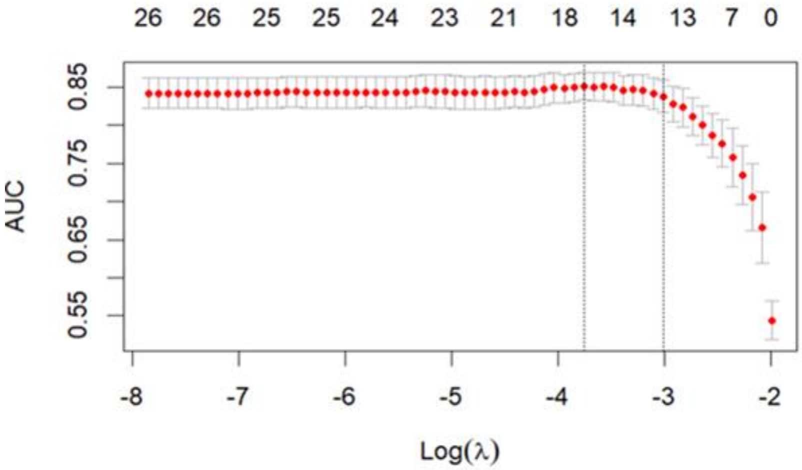

Figure S2. The LASSO regression algorithm was used to select the optimal variable (λ) with a 10-fold cross-validation method

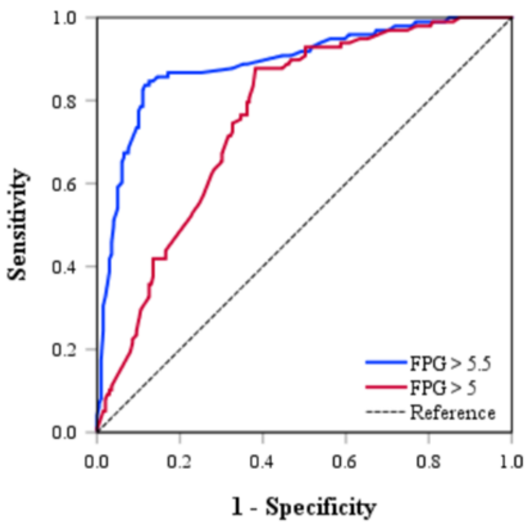

Figure S3. Area of *ROC* curve predicted by Framingham diabetes risk score for different fasting glucose thresholds. The blue curve is the predicted area of the model incorporating FPG>5.5; the red curve is the predicted area of the model incorporating FPG>5.
